# Fasting serum glucose and lymph node metastasis in non-diabetic PTC patients: a 10-Year multicenter retrospective study

**DOI:** 10.7150/jca.71514

**Published:** 2022-05-20

**Authors:** Yushu Liu, Jiantao Gong, Yanyi Huang, Shanshan Xing, Ling Chen, Tao Yi, Zhiyong Wang, Yunxia Lv

**Affiliations:** 1 Department of Thyroid Surgery, Second Affiliated Hospital of Nanchang University, Nanchang, Jiangxi, China.; 2 The second clinical medicine college, Medical Department, Nanchang University, Nanchang, Jiangxi, China.; 3 Department of General Surgery, The First Affiliated Hospital of Jiangxi Medical College, Shangrao, Jiangxi, China.; 4 Department of Otolaryngology, Yichun People's Hospital, Yichun, Jiangxi, China.; 5 Department of Otolaryngology, Xinfeng County People's Hospital, Ganzhou, Jiangxi, China.

**Keywords:** Papillary thyroid carcinoma, Fasting serum glucose, Lymph node metastasis

## Abstract

**Background:** Mostly current studies are limited to the impact of lymph node metastasis(LNM) on the prognosis of papillary thyroid cancer(PTC) or the impact of glucose metabolism on the occurrence of PTC, but no one has paid attention to the connection between fasting serum glucose(FSG) and LNM. The purpose of our study was to explore the relationship between FSG and LNM in non-diabetic PTC patients.

**Methods:** In this study, we performed a multicenter, retrospective study on 6034 non-diabetic patients with PTC. The associations of FSG with three types of LNM including central lymph node metastasis (CLNM), lateral cervical lymph node metastasis (LLNM) and both were estimated.

**Results:** Compared with PTC patients without LNM, those with LNM had higher FSG. We also found that FSG was associated with tumor extension, maximum tumor diameter and TSH. In order to further explore the association between FSG and different types of LNM, we analyzed three groups of data separately. Our study reveals that by comparing FSG between patients without LNM and patients with three LNM types, it was statistically different in the PTC patients with CLNM and the PTC patients with CLNM combined with LLNM.

**Conclusion:** Our study provides evidence for the association of FSG and LNM in non-diabetic PTC patients, with a gradual increase in FSG over the course of the PTC from no lymph node metastasis to CLNM combined with LLNM. Meanwhile, higher FSG is a risk factor for CLNM and CLNM combined with LLNM. In the future, FSG might be used as an indicator for lymph node dissection in PTC patients. However, larger relative studies are needed.

## Introduction

According to the American Cancer Society in 2019, the incidence of thyroid cancer has become the fifth leading cause of cancer in women 1, and the incidence of thyroid cancer has increased faster than any other types of cancer in the world 2, 3. Papillary thyroid cancer (PTC) is the most common type, accounting for 84% of all thyroid cancers 4. Lymph node metastasis (LNM) is one of the accepted prognostic indicators of PTC 5, and compared with lateral cervical lymph node metastasis (LLNM), central lymph node metastasis (CLNM) is more common 6. This is a manifestation of cancer's ability to invade and metastasize. Therefore, the indicators affecting LNM in PTC patients deserve more attention.

The most important three metabolisms in human body are glucose metabolism, lipid metabolism and amino acid metabolism. Any kind of metabolic disorder will affect other metabolisms, which is particularly important in the course of PTC 7. The main forms of sugar in the human body are glucose and glycogen. Glucose is the main form of sugar in blood and it is an important source of energy. The concentration of blood glucose is an important index to reflect the state of glucose metabolism. The oxidation process of glucose can provide energy for cells and promote the growth, development, invasion and metastasis of cells 8. Many researchers have studied the association between diabetes and cancer 9, but according to the data we collected, the role of glucose in the occurrence and development of cancer in PTC patients without diabetes has not been reported. Mostly current studies are limited to the impact of LNM on the prognosis of PTC 10 or the impact of glucose metabolism on the occurrence of PTC 11, but no one has paid attention to the connection between the glucose metabolism and LNM in PTC patients. Therefore, the purpose of our study was to explore the relationship between fasting serum glucose (FSG) and LNM in PTC patients without diabetes.

In this study, we collected the clinicopathological data of 5440 PTC patients from the Second Affiliated Hospital of Nanchang University and 594 PTC patients from The People's Hospital of Yichun City and Xinfeng County People's Hospital for analyzing the relationship between FSG and three types of LNM (CLNM, LLNM, CLNM + LLNM). According to the information we collected, this study is the first to reveal an association between LNM and FSG. In addition, we found that with the course of the PTC from no lymph node metastasis to CLNM combined with LLNM, FSG gradually increased. Higher FSG is a risk factor for CLNM and CLNM combined with LLNM.

## Materials and Methods

### Data sources

In this study, we performed a multicenter, retrospective study on 11073 patients with PTC treated at the Second Affiliated Hospital of Nanchang University from May 2011 to July 2021 and 1336 patients with PTC treated at the Yichun People's Hospital and Xinfeng County People's Hospital from August 2015 to May 2021. The inclusion criteria were histology-confirmed PTC, and complete baseline data (including age and gender), tumor biopsy data (including CLNM, LLNM, number of lesions (hereinafter referred to as 'lesions'), the condition of tumor extension (hereinafter referred to as 'extension'), maximum tumor diameter) and preoperative laboratory data (including preoperative serum TSH, free triiodothyronine (fT3), free thyroxine (fT4) and FSG). The exclusion criteria are as follows: (1) other histological thyroid cancers, such as medullary thyroid carcinoma, follicular thyroid carcinoma and anaplastic thyroid cancer; (2) previously or simultaneously malignant tumor; (3) other thyroid diseases such as hyperthyroidism, hypothyroidism and Hashimoto's thyroiditis; (4) taking thyroid hormone drugs, such as Euthyrox; (5) a disease that affects FSG levels, such as diabetes; (6) taking drugs that affect FSG levels, such as melbine. All patients signed informed consent and were approved by the ethics committee. Screening through exclusion criteria, the remaining 5440 patients from the Second Affiliated Hospital of Nanchang University and 594 patients from the Yichun People's Hospital and Xinfeng County People's Hospital were included in our study.

### Data collection

Baseline data were obtained from outpatient data. Tumor biopsy data were obtained from patient pathology and color Doppler ultrasound reports. All laboratory data (blood chemistry analysis) were acquired in the next morning after the patient's admission to hospital (between 6:00 and 8:00 AM).Patients were required to fast for at least 8 hours before sample collection. The serum was immediately separated.

### Treatment

According to the National Comprehensive Cancer Network guidelines, the standard treatment for our study was thyroidectomy. Laboratory data included preoperative serum TSH, fT3, fT4 and FSG. They were tested through blood samples which were collected from each patient 8 to 10 hours before surgery. An automatic chemiluminescence detection system (Cobas E411) was commonly used machine for the testing.

### Statistical analysis

All statistical analyses were performed using R software (3.6.1). Categorical variables are represented by number and percentage, and continuous variables are represented by mean ± standard deviation. Mann-Whitney U test was used to analyze the difference between continuous variables and Chi-square test was used to analyze the difference between categorical variables. Kolmogorov-Smirnov test was used to analyze the difference among the groups. The Receiver operating characteristic (ROC) curves were used to determine the optimal cut-off value of the variables and the area under the curve (AUC) was used to reflect theirs predictive power. In our study, p-value < 0.05 was statically significant.

## Results

### Clinical baseline characteristics

**Figure 1:**
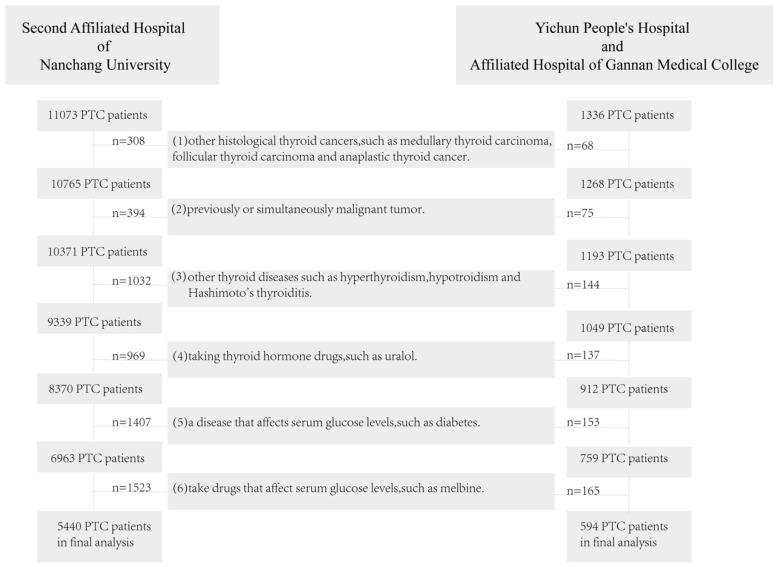
PTC patients exclusion flowchart. **Abbreviation**: PTC, papillary thyroid cancer

**Figure 2:**
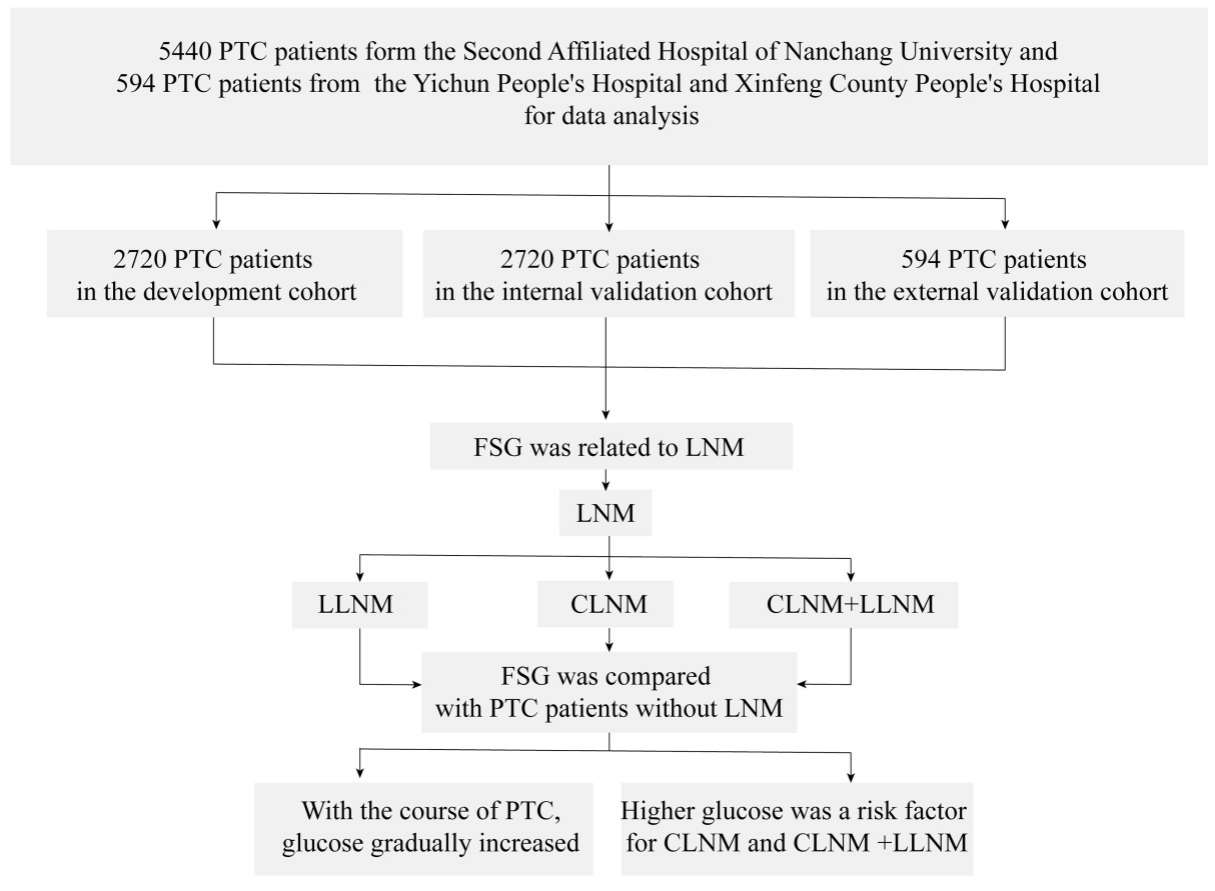
The data analysis process of the article. **Abbreviations**: PTC, Papillary thyroid cancer; FSG, fasting serum glucose; LNM, lymph node metastasis; LLNM, lateral cervical lymph node metastasis; CLNM, central lymph node metastasis

According to the inclusion criteria and exclusion criteria, we finally selected 5440 PTC patients from the Second Affiliated Hospital of Nanchang University and 594 PTC patients from the Yichun People's Hospital and Xinfeng County People's Hospital for data analysis (Figure 1). Our data analysis process is shown in Figure 2.For analyzing the factors which affected LNM in PTC patients, we used the sample function in R to randomly divide the 5440 patients from the Second Affiliated Hospital of Nanchang University into the development cohort (n=2720) and internal validation cohort (n=2720) according to the ratio of 1:1. The patients from the Yichun People's Hospital and Xinfeng County People's Hospital were used as external validation cohort. The development cohort was used to construct the model predicting the status of LNM. The internal and external validation cohorts were used to verify the predictive effect of the model. By using Kolmogorov-Smirnov test 12, we could confirm that there was no statistical difference among three groups. More details were shown in Table 1.

### Explore the association FSG and LNM

**Figure 3:**
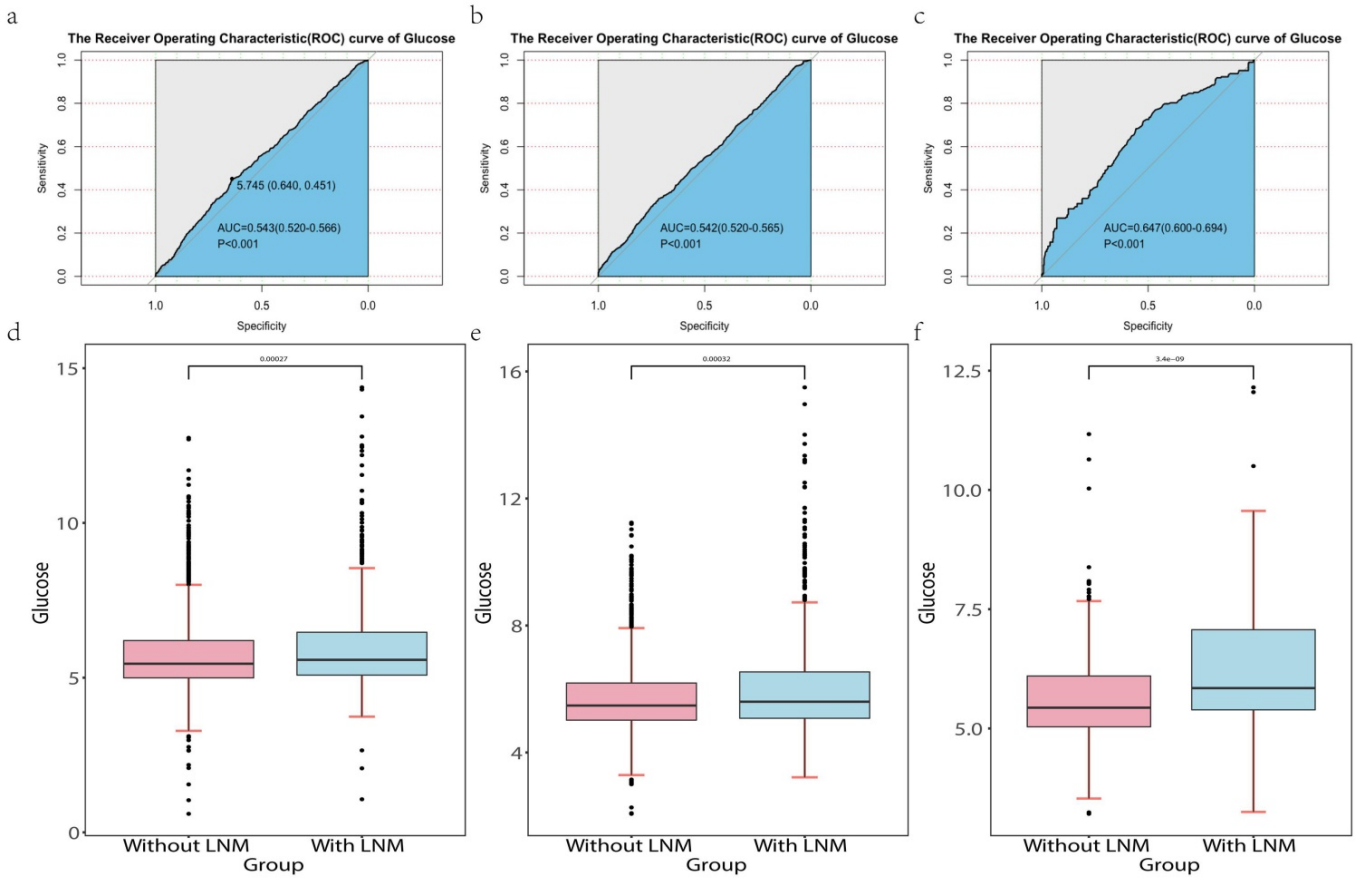
** Abbreviation:** LNM, lymph node metastasis. The Receiver Operating Characteristics (ROC) curve of glucose in the development cohort (a), internal validation cohort (b) and external validation cohort (c).The differential level of glucose in the patients with LNM and without LNM in the development cohort (d), internal validation cohort (e) and external validation cohort (f).

We used the "pROC" package [13]to explore the association between FSG and lymph node metastasis. In our study, P<0.05 was statically significant. The result was shown in Figure 3 a-c. In the development cohort, the AUC and 95%CI of FSG were 0.543 (0.520-0.566, P<0.001). The optimal cut-off values for FSG was 5.745(specificity 64.0%, sensitivity 45.1%).In the internal and external validation cohort, the AUC and 95%CI of FSG were 0.542 (0.520-0.565,P<0.001) and 0.647 (0.600-0.694,P<0.001). It indicates that blood glucose concentration has certain diagnostic value in judging whether lymph node metastasis occurs in PTC patients. In additional, "ggplot2" package 14 was used which visually showed the differential level of FSG in the group with LNM and without LNM in three cohorts (Figure 3d-f). The results revealed that the higher FSG is associated with the greater possibility of LNM. Therefore, higher FSG is an independent risk factor for LNM.

### The correlation between FSG and other indicators

According to the optimal cut-off value in the development cohort, patients were divided into the high and the low FSG groups. In the development cohort, there were statistically significant differences in age (P<0.001), gender (P<0.001), LNM (P<0.001), extension (P<0.001), maximum tumor diameter (P<0.001) and TSH (P<0.001) between the high and the low FSG groups (Table 2).We performed the same analysis in the internal validation cohort and the external validation cohort. In the internal validation cohort, there were statistically significant differences in age (P<0.001), gender (P<0.001), LNM (P=0.010), lesions (P=0.012), extension (P=0.005), maximum tumor diameter (P=0.028) and TSH (P<0.001) between the high and the low FSG groups. In the external validation cohort, there were statistically significant differences in LNM (P<0.001), lesions (P<0.001), extension (P=0.012), maximum tumor diameter (P=0.020) and TSH (P=0.003) between the high and the low FSG groups. In the analysis of the three cohorts, except for lesions, the groups showed statistical differences in LNM, extension, maximum tumor diameter and TSH. It can be seen from the three cohorts that compared with the low FSG group, the incidence of LNM and tumor extension in the high FSG group increased, and the mean TSH also increased. Therefore, it is reasonable to believe that there is some correlation among FSG, TSH, extension, maximum tumor diameter and LNM.

### Effects of FSG on three types of LNM

**Figure 4:**
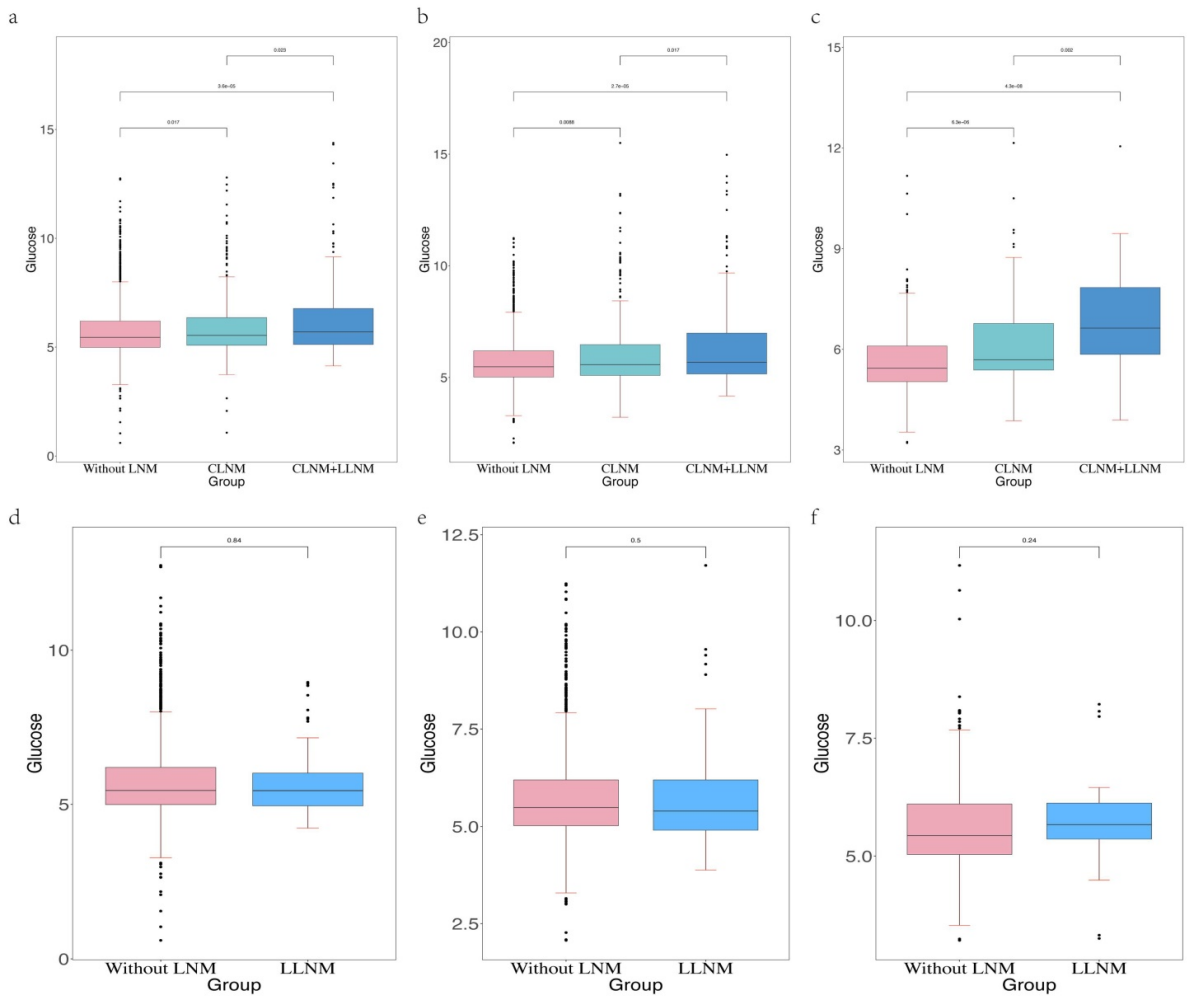
The differential level of glucose in the patients without LNM, with CLNM and with CLNM combined with LLNM in the development cohort (a), internal validation cohort (b) and external validation cohort (c).The differential level of glucose in the patients without LNM, with CLNM and with LLNM in the development cohort (d), internal validation cohort (e) and external validation cohort (f).

Cervical lymph node metastasis (CLNM) in PTC patients is usually considered to first metastasized to the central region of the neck, followed by the lateral neck region 15. However, there is a special condition of LLNM without CLNM. This condition is called skip metastases. The incidence of skip metastases ranged from 6.8% to 27.8% 16, 17. It is consistent with our data (Table 3). Our study has shown that higher FSG is an independent risk factor for LNM, but the predictive power of FSG in the three types of LNM was still not clear. Therefore, in order to further determine whether FSG also has strong predictive power among the three LNM types and in which types of LNM it has the best predictive power, we compared the distribution of FSG between patients without LNM and patients with three LNM types (Figure 4). We found that higher FSG could be a risk factor for CLNM and CLNM combined with LLNM. The occurrence and location of LNM can be seen as one of the diagnostic criteria for the course of cancer. In the three groups of patients without LNM, only with CLNM, and with CLNM combined with LLNM, the FSG value of patients without LNM was the lowest, and the FSG value of patients with CLNM combined with LLNM was the highest. And there was a statistically significant difference in FSG. Therefore, we believed that the FSG gradually increased with the course of PTC. Clinically, when patients have high blood glucose manifestations, we are supposed to be alert to the occurrence of CLNM and CLNM combined with LLNM. Central lymph node dissection can be performed if necessary. However, there was no statistical difference in FSG between patients without LNM and patients with skip metastasis, this may be because skip metastasis is mainly affected by the location and size of the primary tumor. According to Dou Y et al. 18, PTC patients are more prone to skip metastasis when their tumors are located at the upper pole of the thyroid gland. In addition, skip metastasis is more likely to occur when the diameter of primary tumor is less than 0.5cm 19. It can be inferred that skip metastasis is more likely to occur early in the course of PTC.

## Discussion

LNM is a significant risk factor affecting the prognosis of cancer patients 5, so it is very useful to explore the related factors of LNM. Glucose is an important factor reflecting human metabolic status 20, but no one has analyzed the correlation between FSG and LNM yet. For the first time, we analyzed the association between FSG and LNM in non-diabetic PTC patients. In this study, we analyzed it from 2,720 PTC patients spanning 10 years and validated our result in both an internal validation cohort of 2,720 PTC patients and an external validation cohort of 594 PTC patients. We drew a conclusion that FSG and LNM are indeed correlated to a certain extent. FSG gradually increased throughout the progression of PTC from no lymph metastasis to LLNM (excluding skip metastasis). Meanwhile, higher FSG is a risk factor for CLNM and CLNM combined with LLNM. The long time span and large number of patients enhance the reliability of the results of our study.

Others have studied the effects of many factors on LNM 21-23. Compared with previous studies, the current work is a significant advance. Prior studies were limited to data from a single institution and were not validated by external data. More importantly, no studies have been conducted on the relationship between FSG and LNM in PTC patients without diabetes. Our study not only included multiple cohorts from three institutions for training and validation, but also for the first time analyzed the association between FSG and LNM in PTC patients without diabetes and strictly screened eligible patients. Furthermore, we analyzed PTC patients without lymph node metastasis, only with CLNM, CLNM combined with LLNM and skip metastasis, and found that FSG increased over the course of the PTC from no lymph node metastasis to CLNM combined with LLNM.

Our data confirm previous studies. Xiang Y et al. 24 showed that elevated TSH is a risk factor for LNM, which may be related to the ability of LncPVT1 to regulate the proliferation of thyroid cancer cell by recruiting EZH2 and regulating thyroid stimulating hormone receptors 25. Lower fT3 concentration^26^ is associated with increased thyroid cancer, and its bioavailability is regulated by three pyiodimidine deiodinase (D) [type I(D1), type II(D2), and type III(D3)] 27. Maximum tumor diameter 28, lesions 29 and extension 6 are visual representations of tumor growth, which are also reported to be closely related to LNM. Existing studies 18, 30 have claimed that tumor site, maximum tumor diameter, gender, extension and CLNM are risk factors for LLNM, but the association between FSG and it has not been reported.

Our study was the first to find an association between FSG and LNM, with a gradual increase in FSG over the course of the PTC from no lymph node metastasis to CLNM combined with LLNM. Meanwhile, higher FSG is a risk factor for CLNM and CLNM combined with LLNM.

TSH is considered to be a major stimulant of thyroid function 31 and cell proliferation 32. TRH stimulates the pituitary and promotes the synthesis and secretion of TSH. TSH acts on the thyroid gland and stimulates thyroid hormone biosynthesis and secretion. T3 and T4 regulate the secretion of TRH and TSH through negative feedback 33. TSH induces thyroid cell growth directly by binding to its own receptors, and indirectly by stimulating the production of autocrine or parocrine growth factors such as vascular endothelial growth factor (VEGF) 34 and amyloid precursors 35. VEGF-C induces lymphangiectasia (lymphangiogenesis) in primary tumors and drained sentinel lymph nodes to promote the growth of tumor-associated lymphatic vessels and enhance LNM 36. But other pathways such as mitogen-activated protein kinase (MAPK) 37, phosphoinositide 3 kinase (PI3-K) 38, mammalian target of rapamycin (mTOR) 39 and insulin growth factor (IGF) system 40 play an equally important role in the proliferation and growth of thyroid cells and their precursors/stem cells.

Insulin /IGF axis is an important pathway for the proliferation of normal thyroid cells and tumor cells. In addition to its own effects, the stimulation of TSH on thyroid growth is partially dependent on other growth hormones, including insulin and IGF 41. They coordinate with cAMP to regulate the expression of thyroid transcription factor 2(TTF-2) 42. Studies have shown that crosstalk between insulin/IGF axis and TSH also plays a role in abnormal thyroid cell proliferation, and insulin and IGF can greatly enhance the tumor-promoting effect of TSH 43. At the same time, stimulation of TSH on FRTL-5 thyroid cells enhances tyrosine phosphorylation of insulin receptor substrate (IRS)-2 triggered by IGF-1, leading to enhanced IGF-1-dependent proliferation 44.

Overexpression of IGF-1, IGF-1R, IGF-2 and insulin receptor (IR) can be seen in the early stage of thyroid cancer 45. Insulin-like growth factor-1 receptor (IGF-1R) plays an important role in diabetic insulin resistance and hyperinsulinemia, and it has been reported that the expression of IGF-1R is significantly increased in lung cancer combined with type 2 diabetes 46. Yan Y et al. 47 showed that in PTC tissues, the expression of IGF-1R in diabetic patients was significantly higher than that in non-diabetic patients. Hyperglycemia can promote the production of advanced glycation end products (AGEs), and then tumor cell proliferation, and directly or indirectly promote IGF-1R phosphorylation (activation) 48, 49. Elevated circulating insulin levels are associated with an increased risk of cancer and aggressive and metastatic cancer phenotypes 50. Our study showed that in PTC patients without diabetes, higher FSG was positively associated with cancer progression, and hyperglycemia was strongly associated with an increased maximum tumor diameter and extension, which may also be associated with IGF-1-associated insulin resistance and hyperinsulinemia. However, more data is needed to prove our conjecture. Whether IGF-1 is activated in PTC patients without diabetes and the specific mechanism and pathway of activation also need more research and demonstration.

The advantage of our study is that for the first time, we found a potential association between FSG and LNM. Based on a large amount of clinical data and a long time span, FSG increased over the course of the PTC from no lymph node metastasis to CLNM combined with LLNM. Higher FSG is a risk factor for CLNM and CLNM combined with LLNM. However, our study still has some shortcomings. First, the retrospective nature of the study may cause bias. Meanwhile, the patients included in our study were limited to Jiangxi Province, and patients from other regions of China or other countries were not studied. Regional environmental differences and ethnic differences may also cause certain bias. Our data span 10 years, which can enhance the generalizability of the analysis results, but given the update of testing technology during the decade, different testing instruments may bias the analysis results. In addition, there are technical limitations in detecting CLNM, so the diagnosis of skip metastases may not have high sensitivity and specificity. The lack of prospective studies to validate the prediction model is a major limitation of this study. In spite of this, the LNM prediction model established by us still has satisfactory predictive power, and the increase of FSG with the course of the PTC from no lymph node metastasis to CLNM combined with LLNM is credible.

## Conclusion

Our results suggest that FSG have a good ability to predict LNM in PTC patients, with a gradual increase in FSG over the course of the PTC. Meanwhile, higher FSG is a risk factor for CLNM and CLNM combined with LLNM. However, prospective studies and more data are needed to confirm our conclusion.

## Figures and Tables

**Table 1:** Baseline characteristics of PTC patients

Charateristics	Development cohort	Internal validation cohort	External validation cohort	P
	N/Mean±SD			
**Number of patients**	n = 2720	n = 2720	n = 594	-
Age	42.72±11.74	43.11±11.95	43.56±12.93	0.253
**Gender**				
Male	676(24.85%)	646(23.75%)	135(22.73%)	0.443
Female	2044(75.15%)	2074(76.25%)	459(77.27%)	
**LNM**				
Yes	918(33.75%)	921(33.86%)	208(35.02%)	0.836
No	1802(66.25%)	1799(66.14%)	386(64.98%)	
**Clnm**				
Yes	852(31.32%)	838(30.81%)	182(30.64%)	0.899
No	1868(68.68%)	1882(69.19%)	412(69.36%)	
**LLNM**				
Yes	312(11.47%)	295(10.85%)	67(11.28%)	0.762
No	2408(88.53%)	2425(89.15%)	527(88.72%)	
**Lesions**				
Unifocal	1934(71.10%)	1882(69.19%)	405(68.18%)	0.187
Multifocal	786(28.90%)	838(30.81%)	189(31.82%)	
Extension				
Yes	646(23.75%)	683(25.11%)	141(23.74%)	0.471
No	2074(76.25%)	2037(74.89%)	453(76.26%)	
**Maximum tumor diameter (cm)**	1.16±0.96	1.12±0.93	1.22±1.03	0.115
**FSG**	5.81±1.32	5.84±1.33	5.82±1.17	0.239
**TSH**	2.22±2.48	2.17±2.29	2.18±2.14	0.275
**fT3**	3.23±0.52	3.25±0.56	3.13±1.01	0.170
**fT4**	1.28±0.31	1.29±0.37	1.28±0.38	0.516
**fT3/fT4**	2.59±0.50	2.61±0.55	2.63±1.16	0.148

**Abbreviations**: PTC, papillary thyroid cancer; LNM, lymph node metastasis; CLNM, central lymph node metastasis; LLNM, lateral cervical lymph node metastasis; TSH,t hyrotrophin; fT3, free triiodothyronine; fT4, free thyroxine; extension, extension of tumor

**Table 2:** Correlation between FSG and other indicators of PTC patients in the development cohort,internal and external validation cohort

Characteristics	Development cohort			Internal validation cohort			External validation cohort			
	FSG<5.745 (n=1658)	FSG≥5.745 (n=1062)	P	FSG<5.745 (n=1624)	FSG≥5.745 (n=1096)	P	FSG<5.745 (n=359)	FSG≥5.745 (n=235)		P
**Age (years)^#^**	40.97(11.5)	45.46(11.6)	<0.001^*^	41.58(11.9)	45.39(11.6)	<0.001^*^	42.81(12.9)	44.71(12.9)	0.073	
**Gender**										
Male	369(22.3%)	307(28.9%)	<0.001^*^	348(21.4%)	298(27.2%)	<0.001^*^	72(20.1%)	63(26.8%)	0.069	
Female	1289(77.7%)	755(71.1%)		1276(78.6%)	798(72.8%)		287(79.9%)	172(73.2%)		
**LNM**										
Yes	504(30.4%)	414(39.0%)	<0.001^*^	518(31.9%)	403(36.8%)	0.010^*^	101(28.1%)	107(45.5%)	<0.001^*^	
No	1154(69.6%)	648(61.0%)		1106(68.1%)	693(63.2%)		258(71.9%)	128(54.5%)		
**Lesions**										
Unifocal	1198(72.3%)	736(69.3%)	0.107	1154(71.1%)	728(66.4%)	0.012^*^	300(83.6%)	153(65.1%)	<0.001^*^	
Multifocal	460(27.7%)	326(30.7%)		470(28.9%)	368(33.6%)		59(16.4%)	82(34.9%)		
**Extension**										
Yes	353(21.3%)	293(27.6%)	<0.001^*^	376(23.2%)	307(28.0%)	0.005^*^	131(36.5%)	111(47.2%)	0.012^*^	
No	1305(78.7%)	769(72.4%)		1248(76.8%)	789(72.0%)		228(63.5%)	124(52.8%)		
**Maximum tumor diameter(cm)^#^**	1.07(0.8)	1.3(1.1)	<0.001^*^	1.08(0.9)	1.19(1.0)	0.028^*^	1.10(0.9)	1.39(1.2)	0.020^*^	
**TSH^#^**	2.04(2.7)	2.49(2.1)	<0.001^*^	2.02(2.4)	2.39(2.1)	<0.001^*^	2.13(2.3)	2.29(1.8)	0.003^*^	
**fT3^#^**	3.24(0.5)	3.23(0.5)	0.622	3.26(0.5)	3.23(0.6)	0.076	3.08(1.1)	3.21(0.9)	0.093	
**fT4^#^**	1.28(0.3)	1.29(0.3)	0.961	1.28(0.3)	1.29(0.4)	0.967	1.27(0.3)	1.30(0.5)	0.636	
**fT3/fT4^#^**	2.60(0.5)	2.57(0.4)	0.270	2.62(0.6)	2.60(0.5)	0.286	2.58(1.7)	2.69(1.1)	0.327	
										

**Abbreviations:** FSG, fasting serum glucose; PTC, papillary thyroid cancer; TSH, thyroid stimulating hormone; extension, extension of tumor; fT3, free triiodothyronine; fT4, free thyroxine ^#^Mean (standard deviation) ^*^P < 0.05 considered as statistically significant.

**Table 3:** LNM in the development cohort,internal and external validation cohort

LNM	Development cohort (n=918)	Internal validation cohort (n=921)	External validation cohort (n=208)
**CLNM**	606(66.01%)	626(67.97%)	141(67.79%)
**CLNM+LLNM**	246(26.80%)	212(23.02%)	41(19.71%)
**LLNM**	66(7.19%)	83(9.01%)	26(12.5%)

**Abbreviations**: PTC, papillary thyroid cancer; LNM, lymph node metastasis; CLNM, central lymph node metastasis; LLNM, lateral cervical lymph node metastasis.

## Abbreviations

PTC: Papillary thyroid carcinoma
LNM: lymph node metastasis
CLNM: central lymph node metastasis
LLNM: lateral cervical lymph node metastasis
TSH: thyrotrophin
fT3: free triiodothyronine
fT4: free thyroxine
AUC: the area under the curve
CI: confidence interval
ROC: receiver operating characteristic curves
